# ANGPTL3 is a novel HDL component that regulates HDL function

**DOI:** 10.1186/s12967-024-05032-x

**Published:** 2024-03-10

**Authors:** Longyan Yang, Yan Wang, Yongsong Xu, Kun Li, Ruili Yin, Lijie Zhang, Di Wang, Lingling Wei, Jianan Lang, Yanan Cheng, Lu Wang, Jing Ke, Dong Zhao

**Affiliations:** 1 Center for Endocrine Metabolism and Immune Diseases, Beijing Luhe Hospital Capital Medical University, Beijing, China; 2 Beijing Key Laboratory of Diabetes Research and Care, Beijing, China

**Keywords:** Angiopoietin-like protein 3, High-density lipoproteins, Cholesterol efflux, Ant-inflammatory function, Components of HDL

## Abstract

**Background:**

Angiopoietin-like protein 3 (ANGPTL3) is secreted by hepatocytes and inhibits lipoprotein lipase and endothelial lipase activity. Previous studies reported the correlation between plasma ANGPTL3 levels and high-density lipoprotein (HDL). Recently ANGPTL3 was found to preferentially bind to HDL in healthy human circulation. Here, we examined whether ANGPTL3, as a component of HDL, modulates HDL function and affects HDL other components in human and mice with non-diabetes or type 2 diabetes mellitus.

**Methods:**

HDL was isolated from the plasma of female non-diabetic subjects and type-2 diabetic mellitus (T2DM) patients. Immunoprecipitation, western blot, and ELISA assays were used to examine ANGPTL3 levels in HDL. Db/m and db/db mice, AAV virus mediated ANGPTL3 overexpression and knockdown models and ANGPTL3 knockout mice were used. The cholesterol efflux capacity induced by HDL was analyzed in macrophages preloaded with fluorescent cholesterol. The anti-inflammation capacity of HDL was assessed using flow cytometry to measure VCAM-1 and ICAM-1 expression levels in TNF-α-stimulated endothelial cells pretreated with HDL.

**Results:**

ANGPTL3 was found to bind to HDL and be a component of HDL in both non-diabetic subjects and T2DM patients. Flag-ANGPTL3 was found in the HDL of transgenic mice overexpressing Flag-ANGPTL3. ANGPLT3 of HDL was positively associated with cholesterol efflux in female non-diabetic controls (r = 0.4102, p = 0.0117) but not in female T2DM patients (r = − 0.1725, p = 0.3224). Lower ANGPTL3 levels of HDL were found in diabetic (db/db) mice compared to control (db/m) mice and were associated with reduced cholesterol efflux and inhibition of VCAM-1 and ICAM-1 expression in endothelial cells (p < 0.05 for all). Following AAV-mediated ANGPTL3 cDNA transfer in db/db mice, ANGPTL3 levels were found to be increased in HDL, and corresponded to increased cholesterol efflux and decreased ICAM-1 expression. In contrast, knockdown of ANGPTL3 levels in HDL by AAV-mediated shRNA transfer led to a reduction in HDL function (p < 0.05 for both). Plasma total cholesterol, total triglycerides, HDL-c, protein components of HDL and the cholesterol efflux function of HDL were lower in ANGPTL3−/− mice than ANGPTL3+/+ mice, suggesting that ANGPTL3 in HDL may regulate HDL function by disrupting the balance of protein components in HDL.

**Conclusion:**

ANGPTL3 was identified as a component of HDL in humans and mice. ANGPTL3 of HDL regulated cholesterol efflux and the anti-inflammatory functions of HDL in T2DM mice. Both the protein components of HDL and cholesterol efflux capacity of HDL were decreased in ANGPTL3−/− mice. Our findings suggest that ANGPTL3 in HDL may regulate HDL function by disrupting the balance of protein components in HDL. Our study contributes to a more comprehensive understanding of the role of ANGPTL3 in lipid metabolism.

**Supplementary Information:**

The online version contains supplementary material available at 10.1186/s12967-024-05032-x.

## Background

Atherosclerosis-related vascular complications are the main cause of disability and mortality in patients with type 2 diabetes mellitus (T2DM). HDL isolated from the plasma of T2DM patients has consistently been shown to be dysfunctional [1]. HDL possesses multiple protective effects against atherosclerosis and diabetes due primarily to its role in various biological processes including reverse cholesterol transport [2], immunomodulation [3], maintenance of endothelium integrity [4], anti-inflammatory [5] and anti-oxidant [6] effects, and improving pancreatic islet β cell function and insulin sensitivity [7]. At present, the value of HDL-c level alone as a protective factor of cardiovascular disease (CVD) is under dispute [8], since elevated HDL-c levels are not always protective against CVD and have been associated with higher all-cause mortality [9]. It has been proposed that increasing the levels of functional HDL may be beneficial in suppressing atherosclerosis progression. Given the significant role of HDL in metabolic homeostasis and vascular health, there is an urgent need to determine the regulatory mechanisms of HDL function in diabetes.

HDL is a complex and dynamic conglomerate of proteins, lipids and other biomolecules [10]. An increasing number of studies have shown that the protein composition of HDL is a crucial determinant for the multiple protective functions of HDL. HDL is comprised of high abundance proteins such as apolipoprotein A-I (apoA-I), apoA-II, apoC-III and apoE, as well as important low abundance proteins such as lecithin cholesterol acyltransferase (LCAT), cholesteryl ester transfer protein (CEPT), paraoxonase 1 (PON1), sphingosine-1-phosphate (S1P) and serum amyloid A (SAA) [11]. ApoA-I is the most abundant protein in HDL, accounting for approximately 70% of total protein mass, and maintains the structure and function of HDL [12]. Reconstituted HDL containing apoA-I increases cholesterol efflux [13], and improves insulin secretion and glucose clearance in diabetic mice and T2DM patients [14, 15]. S1P has been shown to promote the ABCA1-mediated cholesterol efflux function of HDL [16]. SAA in HDL reportedly impairs HDL-mediated reverse cholesterol transport and is associated with mortality in coronary heart disease patients, as well as in patients with end-stage diabetic kidney disease [17, 18].

Proteomic analyses have identified more than 100 proteins as potential components of HDL [19]. However, one HDL particle does not contain all of these different proteins, suggesting that the function of HDL under specific conditions is related to its protein composition [20]. Using a proteomic approach, Karlsson et al. identified salivary alpha-amylase and alpha-1-antitrypsin as new protein components in HDL isolated from healthy subjects [21, 22], while Vaisar et al. demonstrated that HDL contains complement component 4 and 9, endopeptidase inhibitors, angiotensinogen and plasma retinol–binding protein [23]. Angiopoietin-like protein 4 (ANGPTL4), also known as FIAF, is present in HDL fractions isolated from human and mice plasma and has been associated with HDL dysfunction in T2DM [24, 25].

ANGPTL3 and ANGPTL4, members of the angiopoietin-like protein family, are major inhibitory regulators of lipoprotein lipase activity [26]. ANGPTL3 is exclusively produced by hepatocytes [27]. Previous studies have shown that individuals with loss of function variants in ANGPTL3 exhibited the characteristics of familial combined hypolipidemia including low levels of TGs and LDL-c [28, 29]. In contrast, HDL-c levels did not correlate with ANGPTL3 expression. Studies have shown that HDL-c levels are decreased in subjects with heterozygous loss-off-function variants in ANGPTL3, while others have reported that HDL-c is weakly associated with ANGPTL3 mutations [29, 30]. Therapeutic strategies that downregulate ANGPTL3, thereby reducing TGs and LDL-c in patients with dyslipidemia, have attracted increasing attention [31]. Although the USA Food and Drug Administration (FDA) approved monoclonal antibody targeting of ANGPTL3 for rare cardiovascular indication in 2021, further research is needed on its long-term efficacy and safety [32]. Previously, we have shown that the relationships between plasma ANGPTL3 and HDL-c, apoA-I and HDL cholesterol efflux function were different in non-diabetic individuals and T2DM patients [33]. The relationship between plasma ANGPTL3 and HDL, LDL, total cholesterol, triglycerides, and HDL cholesterol efflux has been reported in previous studies [34–37]. Kraaijenhof et al. demonstrated that ANGPTL3 preferentially bound to HDL in human circulation and affected ANGPTL3 activity [35]. However, it is still unclear whether ANGPTL3 in HDL affects HDL function. In this study, we investigated ANGPTL3 bound to HDL and explored the effect of ANGPTL3 in HDL on the function of HDL, determined the relationship between ANGPTL3 in HDL and other components in human and mice with non-diabetes or type 2 diabetes mellitus.

## Methods

### Study subjects

This study was comprised of 35 patients diagnosed with T2DM and 37 non-diabetic participants recruited from Beijing Luhe Hospital, Capital Medical University. The study complied with the Helsinki Declaration for the investigation of human subjects. The Ethics Committee of Beijing Luhe Hospital approved this study.

The diagnosis of T2DM was based on the 1999 World Health Organization Criteria, which includes random blood glucose ≥ 11.1 mmol/L and/or fasting blood glucose (FBG) ≥ 7.0 mmol/L and/or 2 h blood glucose, during an oral glucose tolerance test (OGTT) ≥ 11.1 mmol/L. The age of the participant ranged between 40 and 70 years. Blood pressure, relevant diseases, and medication information were recorded. Body mass index (BMI) was calculated as described previously [33]. Patients with other types of diabetes, chronic organ failure, cerebrovascular diseases or other chronic disorders, or who were pregnant, or within 1 year of postpartum period, as well as those with infectious diseases, were excluded.

### Clinical and biochemical measurements

Venous blood samples were withdrawn from each patient in the morning after overnight fasting. Sera were separated within 1 h. Routine laboratory measurements were performed including fasting plasma glucose (FBG, mmol/L), insulin, TG (mmol/L), total cholesterol (TC, mmol/L), LDL-c (mmol/L), HDL-c (mmol/L) and uric acid (UA). Pancreatic β-cell function and insulin resistance were measured using the homeostasis model of assessment (Homa-B and HOMA-IR; http://www.dtu.ox.ac.uk/homacalculator/) from the fasting insulin and glucose levels in subjects. Serum samples were stored immediately at − 80 °C until further analysis.

### Measurement of plasma ANGPTL3

The levels of ANGPTL3 in the plasma were measured using the Human ANGPTL3 Assay Kit (IBL International GMBH, Japan) according to the manufacturer's instructions.

### HDL preparation by ultracentrifugation

Plasma samples were separated by density gradient ultracentrifugation in a swing-out rotor as described previously [25]. Based on the density, very low-density lipoprotein/intermediate-density lipoprotein (VLDL/IDL; g < 1.019 g/mL), LDL (1.019 g/mL < g < 1.063 g/mL), and HDL (1.063 g/mL < g < 1.21 g/mL) fractions were isolated.

### HDL isolation

A Cholesterol Assay Kit (ab65390) was used to separate HDL and LDL/VLDL fractions according to the manufacturer’s instructions. Plasma samples were mixed with precipitation buffer, incubated for 10 min, and centrifuged for 10 min at 2000×*g*. The supernatant was collected.

### Quantification of HDL components by ELISA

The concentrations of apoA-I, phospholipid (PL), S1P and SAA in HDL fractions were measured using the corresponding ELISA kits according to the manufacturer’s instructions (MLBio, Shanghai, China).

### Cell culture

RAW264.7 macrophages and endothelial cells were purchased from the National Infrastructure of Cell Line Resources (Beijing, China). RAW264.7 macrophages were cultured in DMED medium (Gibco, Cleveland, TN, USA) with 10% fetal bovine serum (FBS; Gibco) and 1% penicillin/streptomycin in a 37 °C/5% CO2 incubator. Endothelial cells were cultured in DMED medium (Gibco) with 20% FBS (Gibco) and 1% NEAA in a 37 °C/5% CO2 incubator. When reached about 80% confluence, cells were digested with 0.25% trypsin–EDTA solution and dispensed into new culture flasks at a subcultivation ratio of 1:3.

### Cholesterol efflux capacity measurement

Cholesterol efflux capacity was detected as described previously [33]. Briefly, macrophages (RAW264.7) were plated at a density of 1 × 10^5^ cells/well in a white 96-well plate. After adherence for 2 h, cells were incubated with fluorescent-labeled cholesterol for 16 h. Cells were washed with PBS and then exposed to 100 μg/mL HDL for 4 h. The supernatant was transferred to a new white 96-well plate, and cells were lysed using 100 μL lysis buffer. The fluorescence intensity of the supernatant and cell lysate were measured (Ex/Em = 482/515 nm). The cholesterol efflux capacity was calculated using the following formula: Cholesterol efflux % = Media/(Cell lysate + Media)*100.

### Analysis of anti-inflammatory function by flow cytometry

Briefly, endothelial cells were plated at a density of 2 × 10^5^ cells/well in a 12-well plate, and allowed to attach for overnight. Endothelial cells were incubated with HDL (100 μg/mL) isolated from mice plasma, and then exposed to 10 nM TNF-α for 16 h. The cells were washed twice with PBS (2 mL/tube), centrifuged at 2000 rpm at 4 °C for 5 min, then the supernatant was removed. Cells were stained with FITC mouse anti-human VCAM-1 (1:100) and PE mouse anti-human ICAM-1 (1:100) (BD Biosciences) antibodies for 30 min on ice in the dark. After washing with PBS, the expression of VCAM-1 (FITC) and ICAM-1 (PE) in the cells was analyzed by flow cytometry using the FACS BD II ARIA cytometer. Data were analyzed with FlowJo software V.10.1 (Tree Star, Ashland, OR, USA).

### Experimental animals

The model of mice with overexpressed or knockdown ANGPTL3 were established by tail vein injection of adeno-associated virus. The AAV2/8 overexpressed or down-expressed ANGPTL3 the corresponding controls were purchased from Vigene Biosciences (Jinan, China). In brief, Db/db mice (6–8 weeks old) were allowed to adapt to their new environment for 1 week, and then db/db mice were intravenously injected with AAV2/8 overexpressing or knocking down ANGPTL3 at a dose of 2.5*10^11^ vg. Eight weeks post-injection, mice were fasted overnight and anesthetized with isoflurane, and collected serum and liver for further study.

### Generation of ANGPTL3-knockout mice

ANGPTL3 knockout mice (ANGPTL3−/−) were generated by Beijing Viewsolid Biotechnology Co., Ltd (Beijing, China). Genotyping of wild-type and knockout mice was performed with the following primers: forward primer 5′-CCCATTCCTATCATAAATGGAGG-3′ and reverse primer 5′-CCCATTCCTATCATAAATGGAGG-3′ for wild-type mice; and forward primer 5′-AGTGCCTATTAGACAGCAAGAAAG-3′ and reverse primer 5′-GAGAAACGACACCCTTCACAG-3′ for ANGPTL3−/− mice. C57BL/6 mice were used and maintained under specific pathogen-free conditions.

### Lipid determination in mice

TC, TG, HDL-c and LDL-c levels were assayed in plasma samples using a Chemray 800 clinical analyzer (Shenzhen, China).

### Western blotting

Protein lysates were separated on 10% SDS‑PAGE and transferred onto polyvinylidene difluoride membranes (0.45 μm). Membranes were blocked in 5% non-fat dry milk in TBS + 0.05% Tween-20 (TBST) buffer for 1 h at room temperature, then incubated with primary antibodies overnight at 4 ˚C. Membranes were washed four times for 5 min with TBST buffer and incubated with a horseradish peroxidase (HRP)-conjugated anti-mouse IgG or anti-rabbit IgG secondary antibody (1:3000; TA130001 and TA130015, OriGene Technologies) for 1 h at room temperature. Membranes were washed four times for 5 min with TBST buffer and visualized using an Electrochemiluminescence (ECL) Kit (Applygen Technologies). Protein bands were analyzed using ImageJ software (Version 1.80, NIH, USA).

### Quantitative real-time PCR (qRT-PCR)

Total RNA was extracted using TRIzol (Invitrogen, USA) according to the manufacturer’s instructions. A HiScript® II 1st Strand cDNA Synthesis Kit was used to obtain the cDNA sequence (Vazyme Biotech, Nanjing, China). mRNA expression levels were quantified using SYBR Green Supermix (Bio-Rad, CA, USA) according to the manufacturer's protocol. The following primers were used: 18s: Forward: GTAACCCGTTGAACCCCATT, Reverse: CCATCCAATCGGTAGTAGCG; and ANGPTL3: Forward: GAGGAGCAGCTAACCAACTTAAT, Reverse: TCTGCATGTGCTGTTGACTTAAT.

### Immunoprecipitation

Human plasma HDL was obtained by ultracentrifugation, then the HDL solution was incubated with 1 μg anti-ANGPTL3 and 50 μL protein A-magnetic beads for 1 h with end-over-end rotation at room temperature. After washing four times with PBST buffer, the immunoprecipitated proteins were eluted from the beads with 2X loading buffer and analyzed by western blotting.

### Statistical analysis

Statistical analyses were performed using SPSS 27 software (SPSS Inc., Chicago, IL, USA). Continuous variables were expressed as the mean ± standard deviation (SD). Differences were analyzed using the Student’s t-test or Mann–Whitney test. Pearson or Spearman correlation analyses were used to examine the correlations between ANGPTL3 levels in HDL and metabolic parameters. Multiple linear regression was used to evaluate the multivariate relationships. P values < 0.05 was considered to be statistically significant.

## Results

### ANGPTL3 was a component of HDL in non-diabetic subjects and T2DM patients

**Fig. 1:**
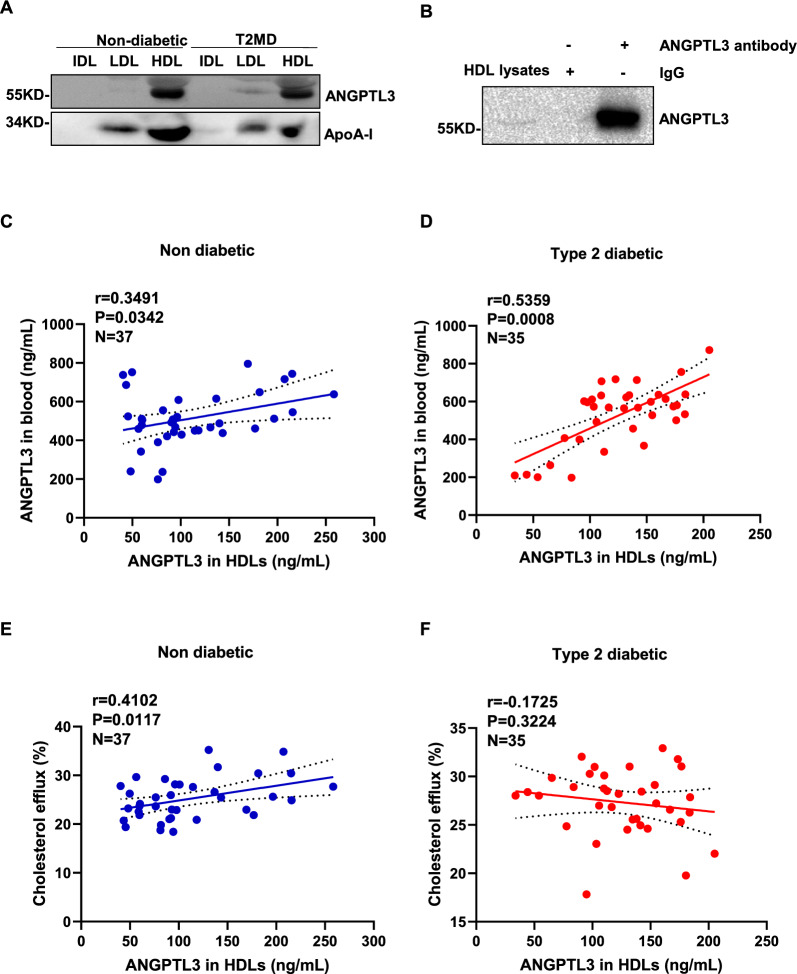
ANGPTL3 was confirmed as a component of HDL in human. **A** ANGPTL3 was present in human plasma HDL. ANGPTL3 was detected in lipoprotein fractions isolated from non-diabetic subjects and type-2 diabetic patients by western blot. **B** HDL contained ANGPTL3. Solubilized lysates from human plasma HDL were subjected to immunoprecipitation with or without anti-ANGPTL3 antibody. The immunoprecipitant complex of ANGPTL3 was probed with anti-ANGPTL3 antibody by Western blot. **C**, **D** The association between the levels of ANGPTL3 in HDL and plasma ANGPTL3 levels. Univariate association between ANGPTL3 in HDL and plasma ANGPTL3 levels in female non-diabetic subjects (**C**) and T2DM patients (**D**). **E**, **F** Association between HDL-ANGPTL3 levels and cholesterol efflux function of HDL. Univariate association between ANGPTL3 in HDL and the percentage of cholesterol efflux in female non-diabetic subjects (**E**) and T2DM patients (**F**)

Previous study suggested that ANGPTL3 preferentially bound to HDL in healthy human circulation and affected ANGPTL3 activity [35]. Lipoprotein fractions were isolated from the plasma of non-diabetic subjects and T2DM patients by density gradient ultracentrifugation. The protein expression levels of ANGPTL3 were measured by western blotting. We found that ANGPTL3 largely bound to HDL, and slightly bound to LDL in non-diabetic subjects and T2DM patients (Fig. 1A). To further verify the presence of ANGPTL3 in HDL, co-immunoprecipitation assays with anti-ANGPTL3 antibody were performed on the HDL lysate of non-diabetic subjects. The immunoprecipitation complexes were examined by western blot analysis using the anti-ANGPTL3 antibody. A robust ANGPTL3 signal was observed in the immunoprecipitation complexes, but no signal was detected in the IgG control immunoprecipitation complexes (Fig. 1B), indicating that ANGPTL3 was present in the HDL particles.

### General characterization of the study subjects

Previously, we have shown that plasma ANGPTL3 levels are associated with HDL components and HDL function in female non-diabetic subjects, but not in male non-diabetic subjects [33]. Thus, we next sought to determine t the relationship between ANGPTL3 in HDL with other HDL components and HDL function in female non-diabetic subjects and T2DM patients. The demographic, clinical and laboratory characteristics of the participants are shown in Table 1. Thirty-seven female non-diabetic subjects were recruited with an average age of 53.92 (5.87) years, BMI of 27.00 (4.42) kg/m^2^, systolic blood pressure of 129.64 (19.18) mm Hg, and diastolic blood pressure of 76.38 (12.00) mm Hg. Twelve of the 37 female non-diabetic subjects (32.43%) had hypertension, while two (5.41%) had coronary heart disease. Thirty-five female T2DM patients were recruited with an average age of 56.60 (5.64) years, BMI of 26.64 (4.33) kg/m^2^, systolic blood pressure of 129.78 (17.26) mm Hg, and diastolic blood pressure of 73.70 (11.04) mm Hg. Twelve of the 35 T2DM patients (34.29%) had hypertension, while none had coronary heart disease. Fasting blood glucose levels were significantly higher in T2DM patients (9.51 (2.78) mg/dL) than non-diabetic subjects (5.04 (0.42) mg/dL) (P < 0.001). Of the T2DM patients, three (8.57%) received insulin injections and 12 (34.29%) used metformin.

**Table 1:** General Characteristics of female non-diabetic participants and type 2 diabetic patients

	Non-diabetic	Type 2 diabetic	P value
n	37	35	
Hypertension (%)	12 (32.43)	12 (34.29)	0.8676
Coronary heart disease (%)	2(5.41)	–	0.1630
Medications (%)			
Lipid-lowering drugs (0,1)	1 (2.70)	–	0.3274
Anti-hypertensive (0,1)	5 (13.51)	3 (8.57)	0.5048
Insulin (0,1)	–	3 (8.57)	0.0689
Metformin (0,1)	–	12 (34.29)	< 0.0001
Mean of characteristics			
Age(year)	53.92 (5.87)	56.60 (5.64)	0.052
Body mass index (kg/m^2^)	27.00 (4.42)	26.64 (4.33)	0.727
Systolic blood pressure (mm Hg)	129.64 (19.18)	129.78 (17.26)	0.974
Diastolic blood pressure (mm Hg)	76.38 (12.00)	73.70 (11.04)	0.328
Fasting blood glucose (mg/dL)	5.04 (0.42)	9.51 (2.78)	< 0.001
Insulin (pmol/L)	58.04 (24.77)	71.41 (42.59)	0.112
Homa-B,%	98.51 (24.85)	38.7 (22.1)	< 0.001
Homa-IR,%	1.10 (0.37)	1.5 (1.0)	0.048
UA (μmol/L)	242.86 (51.56)	241.71 (64.61)	0.381
Total cholesterol (mmol/L)	200.03 (33.86)	204.56 (29.31)	0.546
Triglyceride (mg/dL)	145.61 (78.34)	127.13 (56.25)	0.253
LDL-c (mmol/L)	121.12 (30.76)	127.17 (26.99)	0.379
HDL-c (mmol/L)	55.39 (13.36)	53.43 (10.60)	0.494
HDL components			
Apolipoprotein A‐I (μg/mL)	11.28 (3.61)	9.88 (1.99)	0.045
Serum amyloid A (μg/L)	795.09 (148.91)	1115.40 (285.66)	< 0.001
Phospholipid (mg/dL)	798.07 (310.82)	1001.66 (353.09)	0.011
Sphingosine-1-phosphate (ng/L)	1318.21 (404.95)	2035.57 (864.13)	< 0.001
Triglyceride (mg/dL)	408.13 (67.10)	1054.27 (178.20)	< 0.001

To further examine the differences in HDL components between female non-diabetic subjects and T2DM patients, the concentrations of ANGPTL3, apoA-I, apoA-II, PL, S1P, SAA and TG were detected in plasma HDL by ELISA. The concentration of apoA-I (P = 0.045) was found to be reduced, while levels of S1P, SAA and TGs were found to be significantly increased (P < 0.001 for all) in T2DM patients compared to non-diabetic subjects (Table 1). Plasma ANGPTL3 and HDL components including ANGPTL3 in female non-diabetic controls and T2DM patients have been showed in Additional file 1: Fig. S1.

### Correlation between ANGPTL3 in HDL and HDL function in female non-diabetic subjects and T2DM patients

ANGPTL3 was found to be linearly distributed in HDL in the peripheral blood of female non-diabetic subjects (r = 0.3491, P = 0.0342) and T2DM patients (r = 0.5359, P = 0.0008) (Fig. 1C and D). Then we determined the relationship between ANGPTL3 in HDL and HDL function. The capacity of mediating cholesterol efflux of HDL isolated from non-diabetic subjects and T2DM patients were detected by using macrophages (RAW264.7). ANGPTL3 levels in HDL were found to be positively associated with the percentage of cholesterol efflux in non-diabetic subjects (r = 0.4102, P = 0.0117, N = 37) (Fig. 1E), but not in T2DM patients (r = − 0.1725, P = 0.3224, N = 35) (Fig. 1F).

### Relationship between ANGPTL3 in HDL and lipid metabolic parameters and other HDL components

Since ANGPTL3 is mainly involved in lipid metabolism, we next examined the relationship between ANGPTL3 levels in HDL and metabolic parameters in female non-diabetic subjects and T2DM patients (Table 2). We found that ANGPTL3 levels of HDL were positively correlated with LDL (r = 04045, P = 0.0130), but not correlated with TC, TG and HDL-c in non-diabetic participants. ANGPTL3 levels in HDL did not correlate with any of the lipid metabolic parameters in T2DM patients (Table 2).

**Table 2:** Unadjusted analysis of the relationship between ANGPTL3 in HDLs and metabolic parameters in the study subjects

	Nondiabetic Participants		T2DM	
	r	P Value	r	P Value
Number, n	37		35	
TC (mmol/L)	0.3109	0.0611	− 0.08517	0.6266
TG (mg/dL)	0.01467	0.9313	− 0.2055	0.2363
LDL-c (mg/dl)	0.4045	0.0130*	− 0.06344	0.7173
HDL-c (mg/dl)	− 0.1146	0.4994	0.1219	0.4854
UA(μmol/L)	0.4847	0.0024*	0.2998	0.0802

*P ≤ 0.05

The components in HDL are key determinants of HDL function. Here, we detected the correlation between ANGPTL3 of HDL and other components in HDL using Pearson correlation analyses and found that the correlation between ANGPTL3 in HDL and other HDL components (apoA-I) differed between female non-diabetic subjects and T2DM patients. ANGPTL3 in HDL and apoA-I (r = 0.88, P < 0.0001) were found to be positively correlated in female non-diabetic subjects. In contrast, ANGPTL3 in HDL was negatively associated with apoA-I (r = − 0.40, P = 0.0160) in female T2DM patients (Table 3).

**Table 3:** Relationship between ANGPTL3 in HDLs and other components in HDLs

	Nondiabetic participants		T2DM	
	r	P value	r	P value
Number, n	37		35	
S1P (nmol/L)	0.2130	0.2056	0.2979	0.0822
apoA-I (μg/mL)	0.8770*	< 0.0001	− 0.4041*	0.0160
SAA (μg/mL)	− 0.1834	0.2772	− 0.2437	0.1583
PL (mg/dL)	0.05201	0.7598	− 0.2709	0.1154
TG(mg/dL)	− 0.1492	0.3781	− 0.2959	0.0844

*P ≤ 0.05

### ANGPTL3 was a component of HDL in mice

**Fig. 2:**
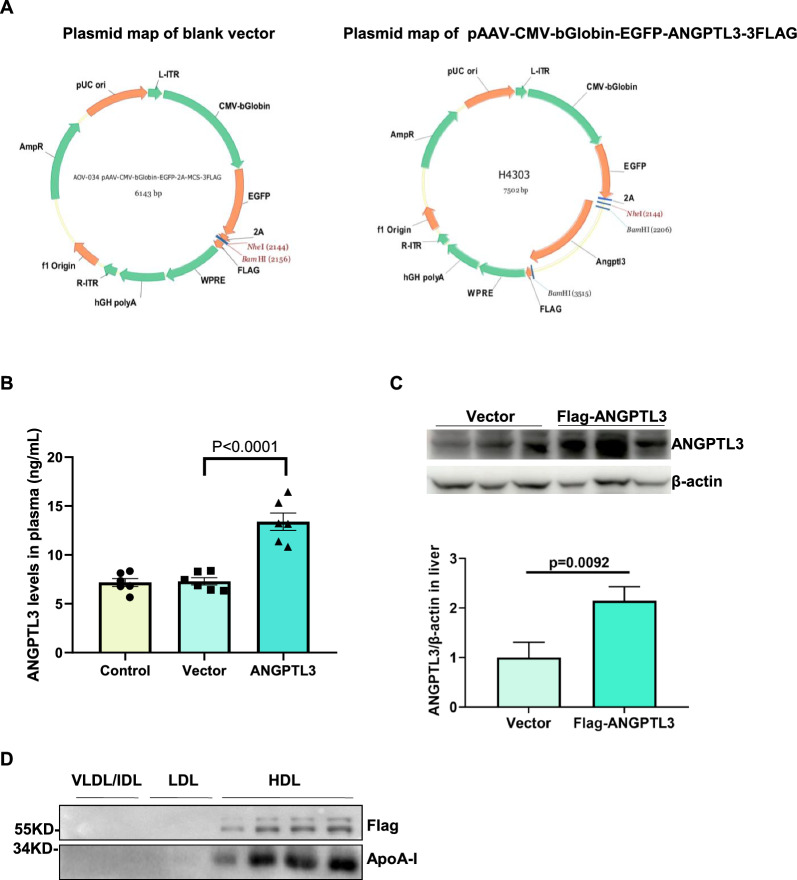
ANGPTL3 was a component of HDL in mice. **A** Schematic structures of AAV2/8-based control vector (AAV-Vector) and AAV-based flag-ANGPTL3 gene vector (AAV-ANGPTL3). **B** ELISA analysis of ANGPTL3 expression in plasma between AAV-Vector and AAV-ANGPTL3 mice. The levels of ANGPTL3 in plasma were detected by ELISA after AAV injection for 8 weeks. **C** The expression of ANGPTL3 in liver tissues between AAV-Vector and AAV-ANGPTL3 mice were analyzed by Western blot. Male C57BL/6 mice were assigned to two groups based on weight. 1*10^11^ virus per mouse were diluted in PBS to a volume of 100 uL and injected intravenously at the tail vein. The ANGPTL3 of liver tissues were detected by Western blot after AAV injection for 8 weeks. **D** SDS-PAGE followed by Western blot confirmed the presence of ANGPTL3 in HDL. The plasma lipoprotein fractions were isolated by ultracentrifugation. Then the fractions were analyzed by Western blot with anti-flag and anti-ANGPTL3 antibodies

To fully confirm the presence of ANGPTL3 in HDL, ANGPTL3 overexpression mice were established. Since ANGPTL3 is exclusively produced by hepatocytes, we used adeno-associated virus 2/8 (AAV2/8), a well-established gene intervention for liver disease [27, 38]. AAV2/8-vector and AAV2/8-ANGPTL3 (cDNA encoding Flag-tagged mouse ANGPTL3 cloned into the AAV2/8-vector) were constructed as shown in Fig. 2A. C57BL/6 mice were injected with either normal saline, AAV2/8-vector or AAV2/8-ANGPTL3 through the tail vein. Eight weeks post-injection, ANGPTL3 levels were found to be increased twofold in the plasma of the AAV2/8-ANGPTL3 group compared to the AAV2/8-vector group (Fig. 2B). ANGPTL3 protein expression levels were still increased by more than twofold in the liver tissue (Fig. 2C). In addition, lipoprotein fractions were isolated from the plasma of AAV2/8-ANGPTL3 mice by density gradient ultracentrifugation and Flag-ANGPTL3 was detected in the HDL by western blotting (Fig. 2D).

### Correlation between ANGPTL3 of HDL and HDL function in mice

**Fig. 3:**
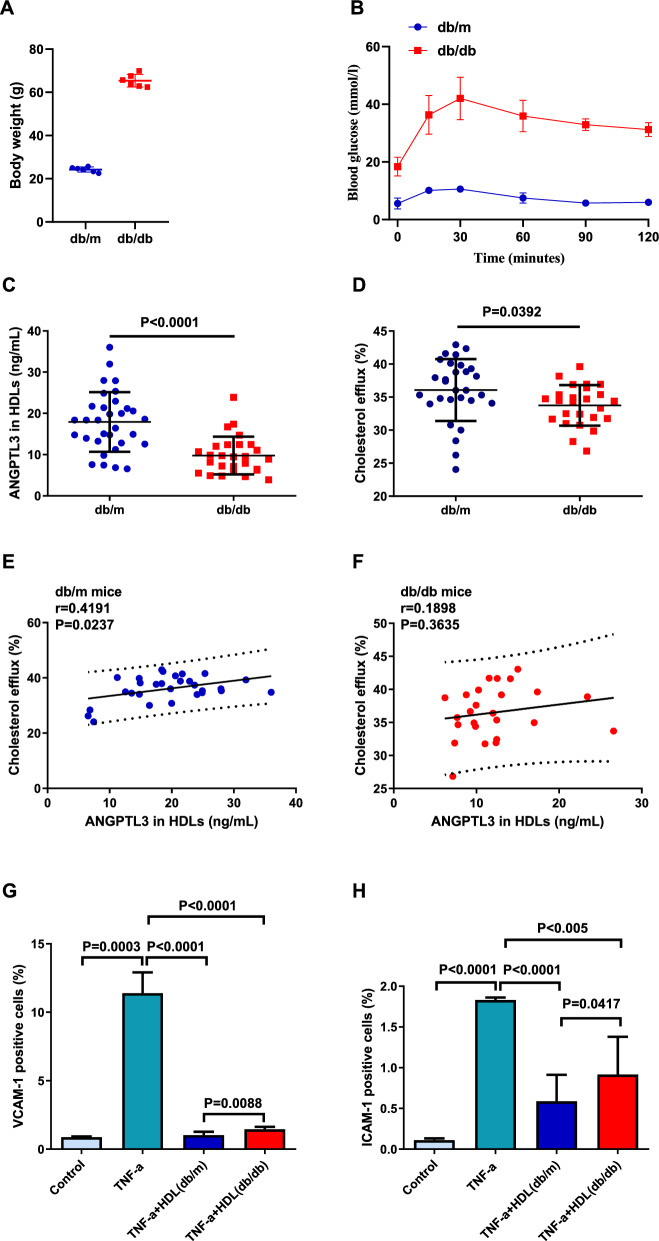
Correlation between ANGPTL3 levels in HDL and HDL function in db/m and db/db mice. **A** Body weight of db/m and db/db mice. **B** Intraperitoneal glucose tolerance tests (IPGTT) in db/m and db/db mice. **C** The ANGPTL3 levels in HDL in db/m and db/db mice. **D** The cholesterol efflux of HDL isolated from db/m and db/db mice. The association between ANGPTL3 in HDL and the percentage of cholesterol efflux in db/m (**E**) and db/db mice (**F**). The expression of ICAM-1 (**G**) and VCAM-1 (**H**) in endothelial cells exposed to TNF-α and pre-incubated with HDL isolated from db/m and db/db mice

The relationship between ANGPTL3 of HDL and HDL function was confirmed in T2DM model mice (db/db) and control mice (db/m). Consistent with previous reports, db/db mice displayed increased weight (Fig. 3A) and impaired glucose tolerance (Fig. 3B). HDL was fractionated from the plasma of db/db and db/m mice at 18 weeks of age. Significantly lower levels of ANGPTL3 were found in the HDL of db/db mice compared to db/m mice (Fig. 3C), while the percentage of cholesterol efflux was decreased in db/db mice compared to db/m mice (Fig. 3D). Next, we analyzed the association between ANGPTL3 in HDL and HDL functions in db/db and db/m mice. We found that ANGPTL3 levels in HDL were positively correlated with the percentage of cholesterol efflux in db/m mice (Fig. 3E), while no correlation was observed in db/db mice (Fig. 3F). The anti-inflammatory function of HDL was assessed by measuring VCAM-1 and ICAM-1 expression levels in endothelial cells pretreated with HDL from db/m and db/db mice, then exposed to TNF-α. A significant increase in VCAM-1- and ICAM-1-postive cells was observed after TNF-α treatment (VCAM-1: P = 0.0003, ICAM-1: P < 0.0001). In addition, VCAM-1 and ICAM-1 expression levels were significantly decreased in the TNF-α + HDL (db/m) group and TNF-α + HDL (db/db) group compared to the TNF-α group, and lower in the TNF-α + HDL (db/m) group than that in TNF-α + HDL (db/db) group (VCAM-1: P = 0.0088, ICAM-1: P = 0.0417) (Fig. 3G and H).

### ANGPTL3 of HDL regulate HDL function in mice

**Fig. 4:**
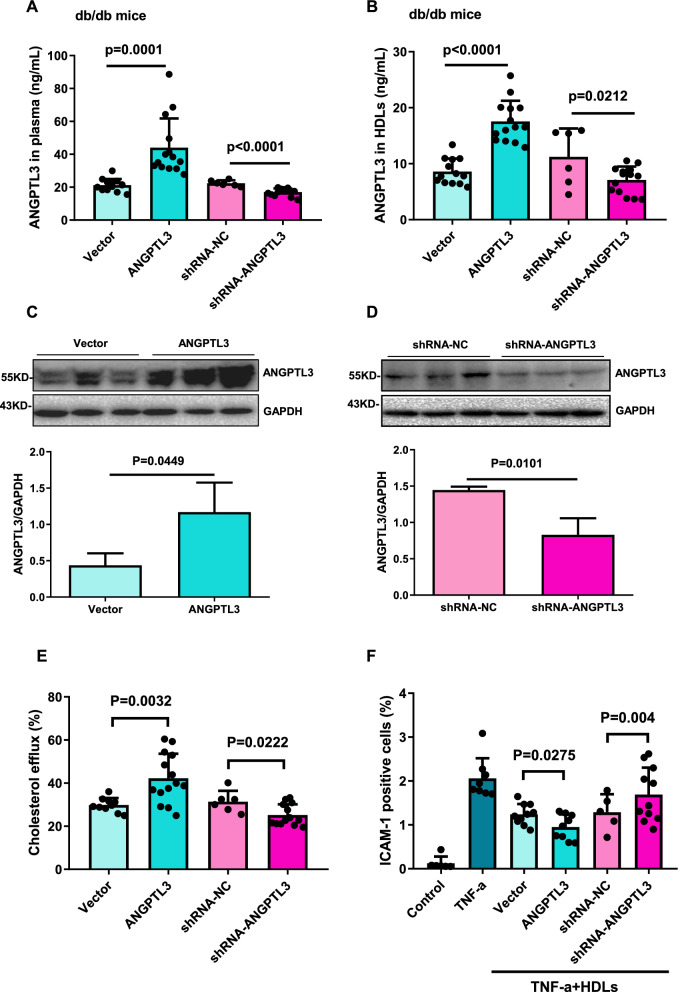
ANGPTL3 directly regulated HDL function in mice. The plasma ANGPTL3 levels (**A**) and HDL-ANGPTL3 levels (**B**) in db/db mice injected with AAV virus encoding vector control, ANGPTL3, shRNA-NC and shRNA-ANGPTL3 plasmid respectively. The expression of ANGPTL3 in liver tissues of db/db mice injected with AAV-Vector and AAV-ANGPTL3 (**C**), AAV-shRNA-NC and AAV-shRNA-ANGPTL3 (**D**). **E** The percentage of cholesterol efflux to HDL isolated from plasma in db/db mice injected with AAV-Vector, ANGPTL3, shRNA-NC and shRNA-ANGPTL3. **F** The expression of ICAM-1 in endothelial cells against HDL. HDL were isolated from plasma in db/db mice injected with AAV-Vector, ANGPTL3, shRNA-NC and shRNA-ANGPTL3. The endothelial cells were treated with HDL, then exposed to TNF-α. The ICAM-1 expression was analyzed by Flow CytoMetry

We next examined the role of ANGPTL3 of HDL in HDL function in db/db mice intravenously injected with AAV2/8 overexpressing or knocking down ANGPTL3. The blood and liver tissue of mice were collected eight weeks post-AAV2/8 administration. ANGPTL3 levels in the plasma and HDL were analyzed by ELISA. We found that overexpression of ANGPTL3 led to a significant increase in ANGPTL3 levels in the plasma and HDL. In contrast, knockdown of ANGPTL3 resulted in a reduction in ANGPTL3 levels in the plasma and HDL (Fig. 4A and B). Western blot analysis was used to measure ANGPTL3 expression levels in the liver tissue. ANGPTL3 expression levels were increased in the ANGPTL3 overexpression (ANGPTL3) group and decreased in the ANGPTL3 knockdown (shRNA-ANGPTL3) group compared to their matched controls (Fig. 4C and D). Overexpression of ANGPTL3 was found to lead to increased cholesterol efflux, while knockdown of ANGPTL3 had the opposite effect (Fig. 4E). In addition, ANGPTL3 overexpression enhanced the inhibitory effect of HDL on TNF-α-induced ICAM-1 expression, while ANGPTL3 knockdown weakened the inhibitory effect (Fig. 4F).

### HDL components and function in ANGPTL3 knockout (ANGPTL3−/−) mice

**Fig. 5:**
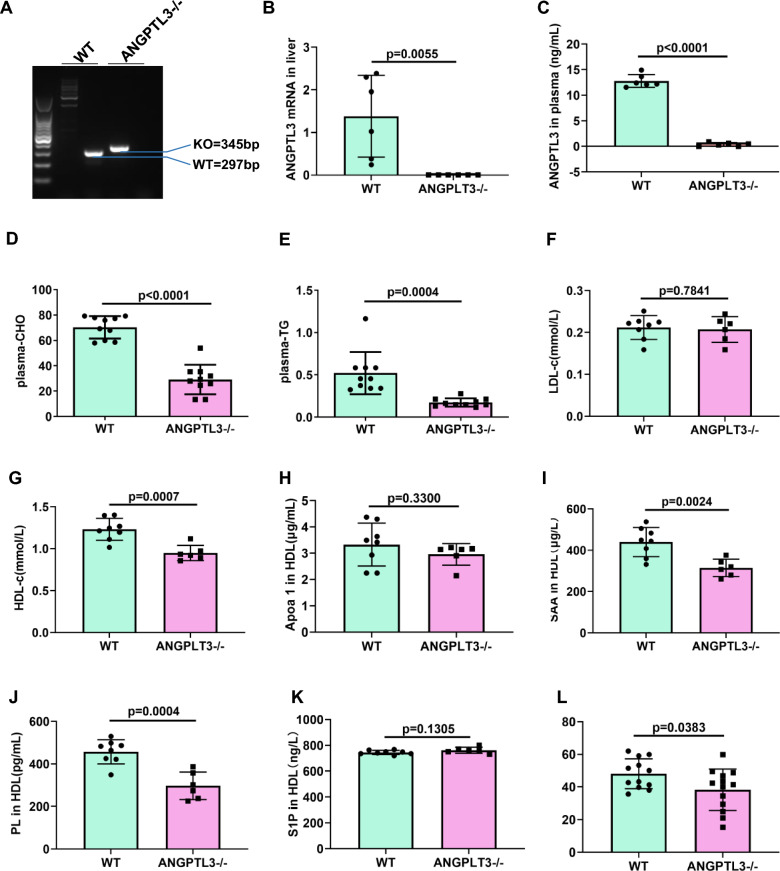
HDL function was decreased in ANGPTL3−/− mice. **A** Phenotype determination of ANGPTL3 knock out mice (ANGPTL3−/−) and wild type mice (WT) in the same nest by RT-PCR. **B** The ANGPTL3 mRNA levels in liver tissues of WT and ANGPTL3−/− mice by RT-qPCR. **C** Plasma ANGPTL3 levels in WT and ANGPTL3−/− mice. The ANGPTL3 expression in plasma of WT and ANGPTL3−/− mice were analyzed by ELISA. D. Total cholesterol levels in plasma of WT and ANGPTL3−/− mice. **E** Total triglyceride levels in plasma of WT and ANGPTL3−/− mice. **F** LDL-c levels in plasma of WT and ANGPTL3−/− mice. **G** HDL-c levels in plasma of WT and ANGPTL3−/− mice. **H** HDL-apoA-I in plasma of WT and ANGPTL3−/− mice. **I** HDL-SAA levels in plasma of WT and ANGPTL3−/− mice. **J** HDL-PL levels in plasma of WT and ANGPTL3−/− mice. **K** HDL-S1P levels in plasma of WT and ANGPTL3−/− mice. **L** The percentage of cholesterol efflux to HDL isolated from plasma in WT and ANGPTL3−/− mice

We constructed ANGPTL3 knockout (ANGPTL3−/−) mice using CRISPR/Cas9 technology. The genotype of ANGPTL3−/− mice and control littermates (ANGPTL3+/+) is shown in Fig. 5A. ANGPTL3 mRNA levels were measured in the liver tissue of ANGPTL3+/+ and ANGPTL3−/− mice (Fig. 5B). Virtually no ANGPTL3 expression was observed in the plasma of ANGPTL3−/− mice (Fig. 5C). TC and TG levels were decreased in the plasma of ANGPTL3−/− mice compared to ANGPTL3+/+ mice (Fig. 5D and E), consistent with previous reports [34, 39]. Furthermore, LDL-c and HDL-c levels were found to be reduced in the plasma of ANGPTL3−/− mice compared to ANGPTL3+/+ mice (Fig. 5F and G). A significant reduction in the levels of the HDL components, apoA-I, SAA, PL and S1P, was observed in HDL isolated from the plasma of ANGPTL3−/− mice compared to ANGPTL3+/+ mice (Fig. 5H–K). Finally, the percentage of cholesterol efflux was found to be decreased in ANGPTL3−/− mice (Fig. 5L).

## Discussion

Recently Kraaijenhof et al. reported that ANGPTL3 predominantly resided on HDL but can also be found on LDL in healthy human subjects, and ANGPTL3 had the highest lipase inhibitory activity when residing on low-density lipoprotein in subjects deficient for HDL [35]. In this study, we confirmed that ANGPTL3 mainly bound to HDL and was a component of HDL in human and mice with non-diabetes or T2DM. In addition, this study is the first to demonstrate the relationship between ANGPTL3 in HDL and HDL function and other HDL components in non-diabetic and T2DM humans and mice. Our key findings include: (1) ANGPTL3was a component of HDL in humans and mice; (2) ANGPTL3 in HDL was positively correlated with apoA-I levels in HDL in female non-diabetic controls but negatively correlated with apoA-I in the HDL of female T2DM patients. ANGPTL3 in HDL was positively correlated with the HDL function of cholesterol efflux in female non-diabetic subjects, while no correlation was observed in female T2DM patients; (3) ANGPTL3 in HDL was positively correlated with the HDL function of cholesterol efflux in db/m mice, while no correlation was found in db/db mice. The anti-inflammatory function of HDL was also weakened in db/db mice compared to db/m mice; (4) Overexpression of ANGPTL3 in HDL promoted cholesterol efflux and the anti-inflammatory function of HDL, while knockdown of ANGPTL3 in HDL reduced HDL functions; and (5) Cholesterol efflux and the protein components of HDL were decreased in ANGPTL3−/− mice compared to ANGPTL3+/+ mice.

ANGPTL3 is produced exclusively by hepatocytes and secreted into the circulation [40]. ANGPTL3 was initially identified from familial hypobetalipoproteinemia in humans due to its role as an inhibitor of both lipoprotein lipase and endothelial lipase [41]. ANGPTL3 has been shown to reduce TG and LDL-c levels, and has therefore emerged as a novel therapeutic target for dyslipidemia [27]. A recent study demonstrated that plasma ANGPTL3 exhibited a negative modulatory function on cholesterol efflux capacity induced by HDL particles [37]. Previously, we have reported that the relationship between plasma ANGPTL3 and HDL-c and HDL cholesterol efflux function was different in non-diabetic individuals compared to T2DM patients [33]. Recently, it’s reported that ANGPTL3 preferentially bounded to HDL in human circulation and affected ANGPTL3 activity [35]. However, it remains unclear whether ANGPTL3 of HDL regulates HDL function in non-diabetic subjects and T2DM patients. ANGPTL3 plays an important role in lipid metabolism and the monoclonal antibody targeting ANGPTL3 has been approved for clinical treatment. Thus, understanding the role of ANGPTL3 on HDL function is critical to provide more comprehensive data for the clinical application of targeted ANGPTL3 drugs. Here, we found that ANGPTL3 largely bound to HDL, and ANGPTL3 slightly bound to LDL. Which was consistent with results previously reported [35]. We confirmed that ANGPTL3 was a component of HDL. HDL components are known to be important determinants of HDL function. ANGPTL4, a member of the same family of proteins as ANGPTL3, has been found in HDL isolated from humans and mice [24]. Furthermore, we have previously shown that ANGPTL4 in HDL protects HDL function [25]. The diverse functions of HDL under different pathophysiological conditions are determined by its complex composition and high heterogeneity [42]. Changes in the concentration of HDL components such as apoA-I, PON1, SAA, and S1P have been shown to directly regulate HDL function [12]. Thus, we hypothesized that as a component of HDL, ANGPTL3 may regulate HDL function.

Loss-of-function mutations in ANGPTL3 have been shown to influence the levels of plasma lipids and lipoproteins [43]. Fazio et al. reported that the effects of plasma ANGPTL3 on plasma lipids and lipoproteins were dependent on the threshold of plasma ANGPTL3 [44]. Plasma ANGPTL3 levels have been shown to correlate strongly and linearly with TC, TG, HDL-c, and LDL-c levels, when plasma ANGPTL3 levels were less than 25% of normal levels. Clinical and animal studies have confirmed that the loss-of-function mutations of ANGPTL3 and antagonists are associated with lower LDL-c levels [45, 46]. However, it is unclear whether ANGPTL3 as a component of HDL is associated with plasma lipids and lipoproteins.

Thus, we next examined the relationship between ANGPTL3 of HDL and metabolic parameters or other components of HDL. We found that ANGPTL3 of HDL was positively correlated with LDL-c but not HDL-c, TC and TGs in female non-diabetic participants, while no correlation between ANGPTL3 of HDL and these lipid metabolic parameters was found in female T2DM patients. Based on these findings, we speculated that ANGPTL3 in HDL might regulate plasma LDL-c levels but not HDL-c levels in female non-diabetic subjects and that the regulatory effect may disappear in female T2DM patients. Previous studies have shown that plasma ANGPTL3 levels were negatively correlated with TC and TG, LDL-c and HDL-c [34–37]. These results suggested that the regulatory effects of plasma ANGPTL3 and ANGPTL3 in HDL on plasma lipids were different. Which is interesting issue, but because of the limited sample size and only female subjects, it could be investigated in our future study.

We found that plasma TC and TG, LDL-c and HDL-c decreased in ANGPTL3−/− mice compared with ANGPTL3+/+ mice, which was consistent with the correlations previously reported between plasma ANGPTL3 and plasma lipids [34, 39, 45]. In ANGPTL3−/− mice, ANGPTL3 in HDL were knocked out but plasma ANGPTL3 also were disrupted. Therefore, these datas were due to results of joint action from plasma ANGPTL3 and ANGPTL3 of HDL.

Although the inverse association between HDL-c and CVD risk is well-documented, more recent clinical trials have questioned the role of HDL-c in determining the risk of developing CVD [47]. Epidemiologic studies in general populations from northern Europe and Canada have shown increased mortality risk at high levels of HDL-c [48, 49]. In addition, the inhibition of CEPT that leads to increased HDL-c levels does not reduce the risk of CVD [50]. Moreover, a prospective, multicenter, cohort study suggested that high HDL-c levels (> 80 mg/dL) were associated with higher mortality risk in subjects with coronary artery disease [51]. Several studies have confirmed that the atheroprotective effects of HDL could be attributed to HDL function, specifically reverse cholesterol transport and its anti-inflammatory capacity, and not HDL-c levels [52, 53].

Since HDL is the highest density lipoprotein (1.063–1.21 g/mL) among plasma lipoprotein classes, the protein content in HDL is also the most abundant, comprising approximately half of total HDL mass [54]. The protein components of HDL play significant roles in HDL function. Here, we clarified that ANGPTL3 of HDL positively regulated HDL function on cholesterol efflux capacity induced by HDL particles. A recent study demonstrated that plasma ANGPTL3 exhibit a negative modulatory function on cholesterol efflux capacity [37]. The role of ANGPTL3 in HDL and plasma ANGPTL3 in HDL function were different. Perhaps ANGPTL3 in HDL affects the content or activity of other components in HDL particles.

We found that overexpression of ANGPTL3 in HDL promoted cholesterol efflux and the anti-inflammatory ability of HDL in db/db mice, while knockdown of ANGPTL3 in HDL reduced HDL function in db/db mice. Our findings suggest that ANGPTL3 in HDL regulates reverse cholesterol transfer and the anti-inflammatory function of HDL in db/db mice. To confirm the direct effect of ANGPTL3 on HDL function, we constructed ANGPTL3 knockout mice and found that cholesterol efflux was decreased in ANGPTL3−/− mice compared to ANGPTL3+/+ mice. In addition, levels of plasma TC and TGs were significantly reduced in ANGPTL3−/− mice, as well as the protein components of HDL. We speculate that ANGPTL3 in HDL regulates HDL function by disrupting the balance of protein components in HDL. Further studies are required to determine how ANGPTL3 in HDL regulates other HDL components.

### Limitations

There are several limitations associated with this study. First, the relationship between ANGPTL3 in HDL and HDL function was only examined in the limited sample size and only female non-diabetic subjects and T2DM patients, based on our previous study that reported that plasma ANGPTL3 levels were associated with HDL components and HDL function in female, and not male, non-diabetic subjects [33]. Second, due to our focus, as well as technical limitations, we did not evaluate the angiogenic function and activity of ANGPTL3. Third, the mechanism by which ANGPTL3 of HDL regulates the other components of HDL was not examined here and will be the focus of future studies.

## Conclusion

We identify ANGPTL3 as a new component of HDL in humans and mice. ANGPTL3 levels of HDL are positively correlated with HDL function including cholesterol efflux and anti-inflammation in non-diabetic subjects, but not in T2DM patients. ANGPTL3 of HDL regulates HDL function in mice. Our findings contribute to a more comprehensive understanding of the role of ANGPL3 in lipid metabolism.

### Supplementary Information

**Additional file 1: Figure S1.** The levels of plasma ANGPTL3 and HDL contents in female non-diabetic controls and type 2 diabetic patients. A. Plasma ANGPTL3 levels in female non-diabetic controls and type 2 diabetic patients. The ANGPTL3 levels in plasma were analyzed by ELISA. B. ANGPTL3 levels in HDLs in female non-diabetic controls and type 2 diabetic patients. HDL from plasma of female non-diabetic controls and type 2 diabetic patients were isolated, then the ANGPTL3 levels in HDLs were analyzed by ELISA. C. Apolipoprotein A-I (apoA-I) levels in HDLs in female non-diabetic controls and type 2 diabetic patients. D. Serum amyloid A (SAA) levels in HDLs in female non-diabetic controls and type 2 diabetic patients. E. Sphingosine-1-phosphate (S1P) levels in HDLs in female non-diabetic controls and type 2 diabetic patients. F. Triglycerides (TG) levels in HDLs in female non-diabetic controls and type 2 diabetic patients. G. Phospholipid (PL) levels in HDLs in female non-diabetic controls and type 2 diabetic patients. **Figure S2.** The levels of HDL-c in db/m and db/db mice. HDL from db/m and db/db mice were isolated, the levels of HDL-c were analyzed separately.

## Data Availability

The datasets supporting the conclusions of this article are included within the article.

## Abbreviations

ANGPTL3: Angiopoietin-like protein 3
HDL: High-density lipoprotein cholesterol
T2DM: Type 2 diabetes mellitus
LDL: Low-density lipoprotein cholesterol
TG: Triglyceride
apoA-I: Apolipoprotein A-I
SAA: Serum amyloid A
S1P: Sphingosine-1-phosphate
PL: Phospholipid
UA: Uric acid
AAV: Adeno-associated virus
VCAM-1: Vascular cell adhesion molecule-1
ICAM-1: Intercellular cell adhesion molecule-1
TNFα: Tumor necrosis factor alpha
