# Supplementary figures and images for: ANGPTL3 is a novel HDL component that regulates HDL function
Source: J Transl Med. 2024 Mar 10;22:263. doi: 10.1186/s12967-024-05032-x (PMC10926621; doi:10.1186/s12967-024-05032-x)

Supplemental figure 1

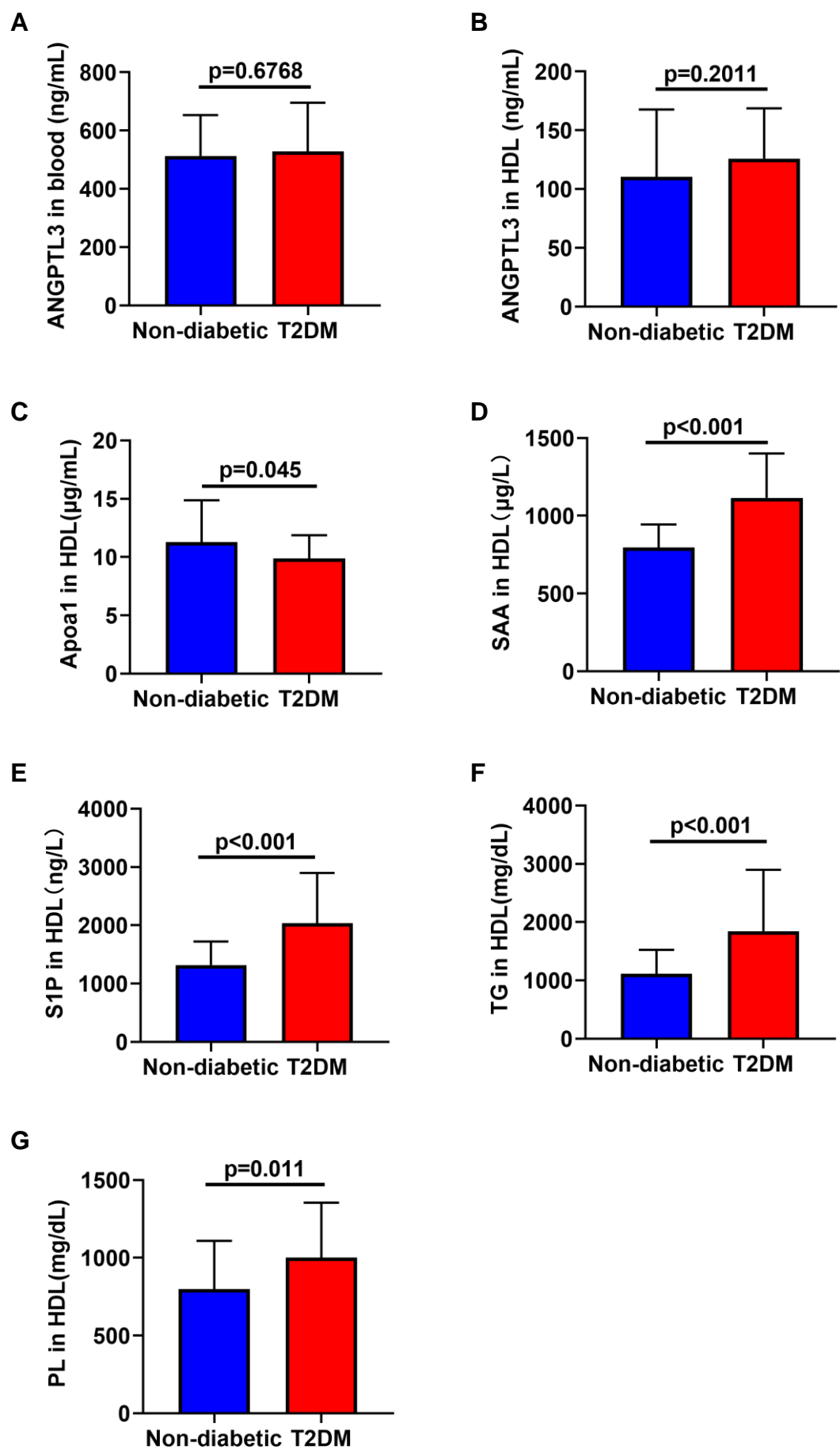

Supplemental figure 2

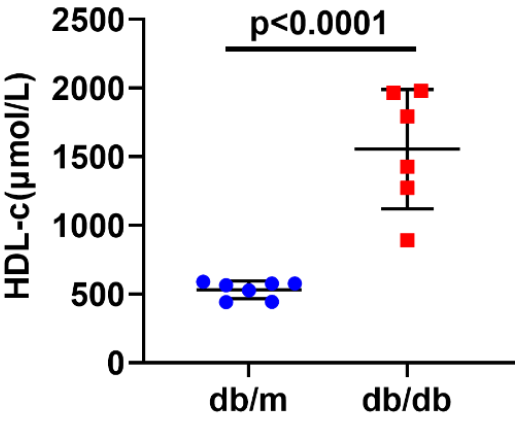

Supplement: Supplementary file 1 — Additional file 1: Figure S1. The levels of plasma ANGPTL3 and HDL contents in female non-diabetic controls and type 2 diabetic patients. A. Plasma ANGPTL3 levels in female non-diabetic controls and type 2 diabetic patients. The ANGPTL3 levels in plasma were analyzed by ELISA. B. ANGPTL3 levels in HDLs in female non-diabetic controls and type 2 diabetic patients. HDL from plasma of female non-diabetic controls and type 2 diabetic patients were isolated, then the ANGPTL3 levels in HDLs were analyzed by ELISA. C. Apolipoprotein A-I (apoA-I) levels in HDLs in female non-diabetic controls and type 2 diabetic patients. D. Serum amyloid A (SAA) levels in HDLs in female non-diabetic controls and type 2 diabetic patients. E. Sphingosine-1-phosphate (S1P) levels in HDLs in female non-diabetic controls and type 2 diabetic patients. F. Triglycerides (TG) levels in HDLs in female non-diabetic controls and type 2 diabetic patients. G. Phospholipid (PL) levels in HDLs in female non-diabetic controls and type 2 diabetic patients. Figure S2. The levels of HDL-c in db/m and db/db mice. HDL from db/m and db/db mice were isolated, the levels of HDL-c were analyzed separately. [file 12967_2024_5032_MOESM1_ESM.pdf]
